# Anterior capsule polishing prevents anterior capsule fibrosis after phacovitrectomy without intraocular lens implantation

**DOI:** 10.1186/s12886-026-04972-w

**Published:** 2026-05-30

**Authors:** Xulong Liao, Qi Zhang, Yuqiang Huang, Weiqi Chen, Andrzej Grzybowski, Haoyu Chen

**Affiliations:** 1 Joint Shantou International Eye Center, Shantou University and the Chinese University of Hong Kong, Shantou, China; 2 Department of Ophthalmology & Visual Sciences, The Chinese University of Hong Kong, Hong Kong, China; 3 Foundation for Ophthalmology Development, Institute for Research in Ophthalmology, Poznan, Poland; 4 Department of Ophthalmology, University of Warmia and Mazury, Olsztyn, Poland

**Keywords:** Anterior capsule polishing, Anterior capsule fibrosis, Phacovitrectomy, Secondary IOL implantation

## Abstract

**Objective:**

To investigate whether anterior capsule polishing can prevent the fibrosis of the anterior capsule in phacovitrectomy without intraocular lens (IOL) implantation.

**Methods and analysis:**

A retrospective study including seventy cases of phacovitrectomy without IOL implantation was conducted. Fifty cases received 360-degree anterior capsule polishing, and 20 cases did not receive polishing. The severity of anterior capsule fibrosis was calculated by adding the score (from 0 to 3) of each clock hour. The fibrosis severity was compared in the groups with and without polishing using Mann-Whitney U test. The risk factors of anterior capsule fibrosis were explored using single and multiple linear regression. The location of secondary intraocular lens implantation was compared between the polishing and non-polishing groups with Chi-square test.

**Results:**

The median severity score of the polishing group was significantly less than that of the non-polishing group (6 vs. 24 out of 36, ***P*** < 0.001). Multiple regression showed that diabetes (b = 14.634, ***P*** = 0.001), silicone oil tamponade (b = 8.734, ***P*** < 0.001), and non-polishing (b = 13.731, ***P*** < 0.001) were the risk factors of anterior capsule fibrosis. Anterior capsule polishing was associated with a higher chance of secondary in-the-bag IOL implantation (OR = 4.889, ***P*** = 0.004).

**Conclusion:**

Polishing the anterior capsule is associated with reduced fibrosis of the anterior capsule in phacovitrectomy without primary IOL implantation, and more chance of secondary in-the-bag IOL implantation.

**Supplementary Information:**

The online version contains supplementary material available at 10.1186/s12886-026-04972-w.

## Introduction

Phacovitrectomy is a common operation for vitreoretinal diseases in patients with cataracts or senile patients [1]. Two options exist for intraocular lens (IOL) implantation, primary or secondary. Primary IOL implantation has the advantage of avoiding additional operation and less cost [2]. However, in patients with retinal detachment, the measurement of biometry is not reliable and may result in IOL power calculation errors [3]. Furthermore, the IOL may limit the visualization of the peripheral retina and bring difficulty in managing the potential peripheral retinal lesions. The other option, leaving the patient aphakic, would cause contact between the anterior capsule and posterior capsule. The residual lens epithelial cells on the anterior capsule would proliferate and lead to anterior capsule fibrosis, manifested as anterior capsule opacification, contraction, and closure of the capsule opening. The opacity of the anterior capsule limits the visualization of the posterior segment, and the closure of the capsule opening makes it difficult for secondary IOL implantation in the bag [4].

In literature, several techniques have been reported to reopen the capsule bag during secondary IOL implantation [5–7]. However, these techniques have a risk of zonule disruption and capsule rupture when manipulating the capsule bag. Some techniques also require special devices [5]. Furthermore, these techniques cannot solve the problem of anterior capsule opacity, which limits the visualization of the posterior segment. To the best of our knowledge, no technique has been reported to prevent anterior capsule fibrosis in aphakic eyes after phacovitrectomy.

Anterior capsule polishing is a surgical technique that removes the lens epithelial cells underneath the anterior capsule [8]. It was first investigated for the prevention of posterior capsule opacification (PCO). However, both literature that supports and against its efficiency on PCO have been reported [9, 10]. It was also reported that anterior capsule polishing can prevent anterior capsule opacity after phacoemulsification and IOL implantation [11]. Therefore, we hypothesize that it can prevent anterior capsule fibrosis after phacovitrectomy without IOL implantation and conducted this retrospective study to validate this hypothesis.

## Methods

This is a retrospective comparative cohort study and follows the tenets of the Declaration of Helsinki. The protocol was reviewed and approved by the Ethics Committee of Joint Shantou International Eye Center of Shantou University and the Chinese University of Hong Kong. Given the retrospective nature of the study and the anonymization of patient data, the requirement for informed consent was waived by the Ethics Committee.

The surgical videos between January 2017 to March 2021 were searched and reviewed. The inclusion criteria were the patients who received phacoemulsification combining pars plana vitrectomy in the first-stage surgical operation, and IOL implantation in the second stage. Exclusion criteria included the patients who had posterior capsule rupture or zonule disruption during phacovitrectomy.

In total, we included 70 eyes of 70 patients. The indications for operation include rhegmatogenous retinal detachment, proliferative diabetic retinopathy and open globe injury involving posterior segment. All surgeries were performed by a single experienced surgeon (HC). The operations were performed under sub-Tenon’s anesthesia. A clear corneal incision was made with a 2.8 mm keratome blade. Viscoelastic was used to inflate the anterior chamber. Continuous curvilinear capsulorhexis at 6 mm diameter, hydrodissection, and phacoemulsification were performed. The cortex was removed with bimanual irrigation/aspiration. In some patients, anterior capsule polishing was performed. Three pars plana incisions were then created at 3 mm posterior to the limbus and vitrectomy was performed. Laser photocoagulation, fluid-air exchange, silicone oil or gas tamponade were performed. IOL was not implanted at this stage.

Two doctors independently reviewed the phacovitrectomy surgical videos and classified the patients into two groups. In the polishing group, 360-degree anterior capsule polishing was performed using bimanual irrigation/aspiration in the polishing mode of the phacovitrectomy system (Constellation, Alcon, Fort Worth, TX) during the first surgery (Supplemental Video 1). While in the control group, anterior capsule polishing was not done. After operation, the patients received topical antibiotics, steroid and cycloplegic.

**Fig. 1:**
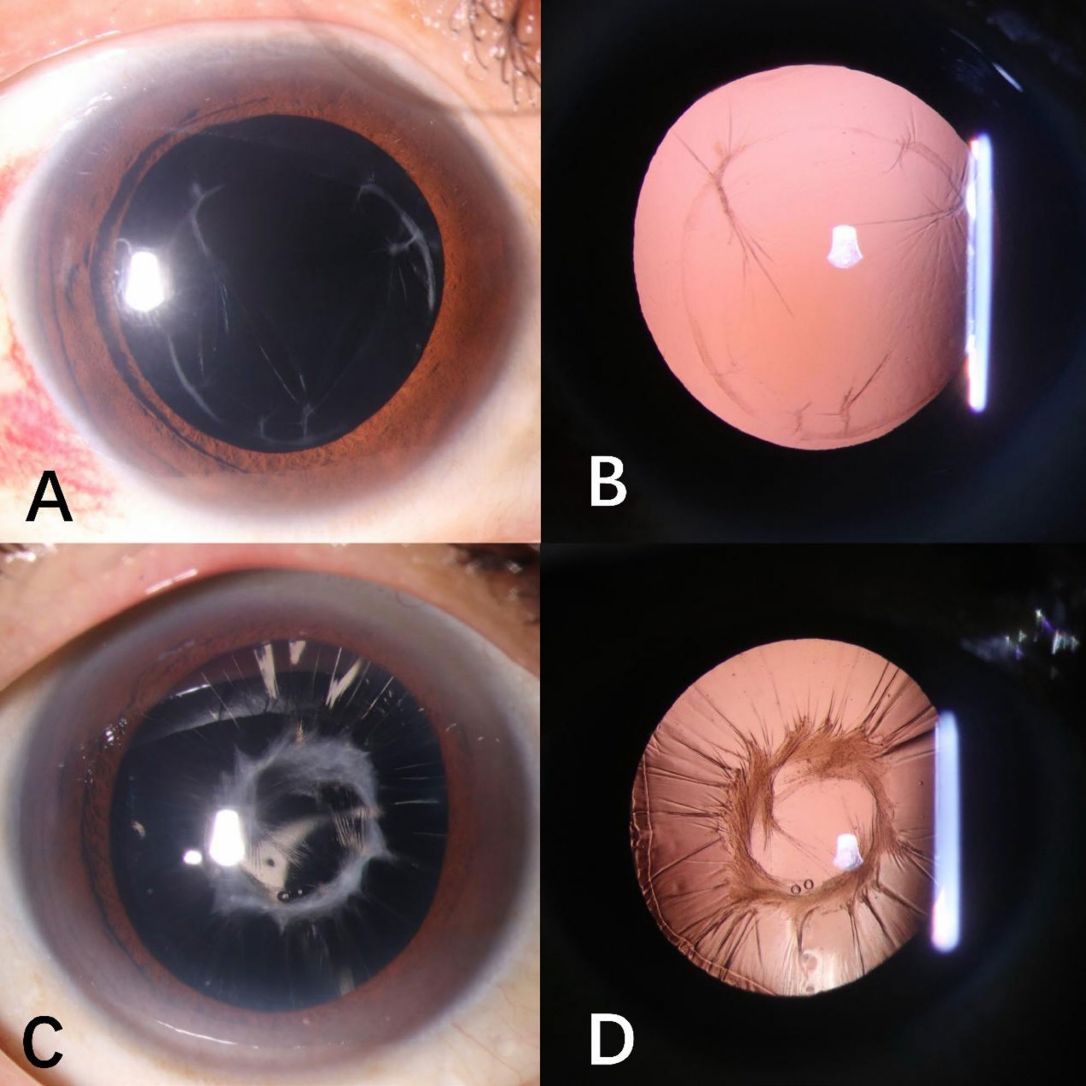
Examples of anterior capsule fibrosis grading after phacovitrectomy. (**A**, **B**): There is mild fibrosis (1 score) at 10, 11 o’clock, moderate fibrosis (2 scores) at 2, 3, 7, 8 o’clock, and no fibrosis (0 score) at other o’clock. The total score is 10. (**C**, **D**): The anterior capsule fibrosis is severe (3 scores) in all 12 o’clock hours and the total score is 36

Intraocular lens implantation was performed in the second stage. The severity of anterior capsule fibrous proliferation was graded independently by two doctors after reviewing the second-stage surgical videos. These assessors were blinded to the information of the first-stage surgery. Because no standardized quantitative scale for anterior capsular fibrosis exists in the current literature, we developed a novel scoring system for this study. The degree of fibrosis at the anterior capsule in each clock hour was graded with four scores (Fig. 1): 0 - no fibrous proliferation at the anterior capsule. 1- fibrous proliferation does not affect the red-light reflection and injection of the viscoelastic agent can directly open the capsule bag. 2 - fibrous proliferation affects the red-light reflection, and the adhesion of the anterior capsule and the posterior capsule can be separated mechanically using an iris repositor or a viscoelastic injection needle. 3 - intensive anterior capsule opacification with folds, and the anterior and posterior capsules cannot be separated due to fibrous proliferation adhesion. The sum of the scores at 12 clock hours was calculated to present the overall severity of anterior capsule fibrosis. The position of IOL implantation was also recorded as in-the-bag implantation and out-of-bag implantation (including sulcus implantation and scleral fixation). Comprehensive ophthalmic examinations were performed for all patients. Best-corrected visual acuity (BCVA) was measured with standard Chinese logarithm visual chart and converted to LogMAR units.

The agreement between the two assessors was analyzed by the kappa coefficient and intraclass correlation. The disagreement on the classification of polishing or non-polishing groups was solved by a senior doctor. The average of the grading between the two assessors was used for further analysis.

The anterior capsule proliferation scores were compared between the polishing and non-polishing groups using the Mann-Whitney U test. We further analyzed the risk factors for the anterior capsule fibrosis using single regression and multi-regression analysis. The position of IOL implantation between the two groups was compared using Chi-square test. The Wilcoxon signed-rank test was used to compare preoperative and postoperative BCVA and data within the same group (intra-group comparison). The Mann-Whitney U test was employed to compare the clinical outcomes between the polishing and non-polishing groups (inter-group comparison).

## Results

A total of 70 eyes of 70 patients were included, and 45 patients (64.3%) were men. The mean age of the patients was 55.2 ± 9.9 years (range 26 to 75 years). All the patients underwent uneventful operations without intraoperative or postoperative complications.

The kappa of classifying polishing and non-polishing groups by the two assessors was 0.833 (95% CI: 0.747–0.894). The intraclass correlation coefficient (ICC) of anterior capsule fibrosis scoring by the two assessors was 0.910 (95% CI: 0.853–0.921).

There are 50 cases received polishing and 20 cases in the non-polishing group. The comparison between the two groups is shown in Table 1. There was no statistically significant difference in gender, age, diabetes, type of tamponade, interval between the two stage operations, axial length, or diagnosis between the two groups. The median score of the polishing group was 6 out of a total of 36 (interquartile range: 2–11), and the median score of the non-polishing group was 24 (interquartile range: 15–32). Mann-Whitney U test results showed that U = 175.0, Z=-4.234, *P* < 0.001.

**Table 1:** Comparison of anterior capsule polishing and non-polishing groups

		Polishing	Non-polishing	*P* value
*N*		50	20	
Gender	Male	32	13	0.937
	Female	18	7	
Age (years, median [IQR]))		55 (50, 63)	57 (50, 61.5)	0.970
Diabetes	Yes	5	0	0.312
	No	45	20	
Tamponade	Oil	23	16	0.248
	Gas	27	4	
Interval between two stages of operation (day, median [IQR])		89 (63, 110)	99 (78.5, 110)	0.427
Axial length (mm, median [IQR])		24.3 (23.1, 26.1)	23.9 (23.5, 25.6)	0.791
Fibrosis score (median [IQR])		6 (2, 11)	24 (14.5, 35)	< 0.001
2nd IOL implantation	In-the-bag	40	9	0.004
	Out-of-the bag	10	11	
Diagnosis	RD	41	17	0.809
	PDR	1	0	
	OGI	8	3	

IOL: Intraocular lens; RD: Retinal detachment; PDR: Proliferative diabetic retinopathy; OGI: Open globe injury

In single regression, diabetes, tamponade, and polishing were identified as associated factors with anterior capsule fibrosis (Table 2). In multi-regression, polishing (b=-13.731, *P* < 0.001), silicone oil tamponade (b = 8.734, *P* < 0.001), diabetes (b = 14.634, *p* = 0.001) were independently associated factors (Table 2).

**Table 2:** Risk factors of anterior capsule proliferation

Factor	Single regression		Multiple regression		
	Coefficient	*P* value	Unstandardized coefficients	Standardized coefficients	*P* value
Gender	0.062	0.609			
Age	0.096	0.428			
Diabetes	**0.286**	**0.017**	14.634	0.316	0.001
Axial length	0.138	0.255			
Interval	0.174	0.149			
Silicone oil tamponade	**0.465**	**< 0.001**	8.734	0.335	<0.001
Polishing	**0.511**	**< 0.001**	-13.731	-0.520	<0.001

In the polishing group, IOL was implanted in-the-bag in 40 (80%) cases, and out-of-the-bag in 10 (20%) cases; in the non-polishing group, IOL was implanted in-the-bag in 9 (45%) cases, and out-of-the-bag in 11 (55%) cases. The odds ratio of unpolishing for out-of-the-bag was 4.889 (95%CI 1.594–11.996), with *P* = 0.004, indicating that 360-degree anterior capsule polishing is helpful for implanting the IOL into the capsular bag in the second stage operation.

Both groups achieved significant visual improvements postoperatively (both ***P*** **< 0.001**; Table 3). Inter-group analysis revealed no significant differences in postoperative BCVA (***P*** **= 0.076**), although the polishing group showed a trend toward better visual acuity (0.40 vs. 0.52 LogMAR).

**Table 3:** Comparison of visual outcomes between groups

Variable		Polishing group (*n* = 50)	Non-polishing group (*n* = 20)	Inter-group *P* value
BCVA (LogMAR)	Preoperative	1.12 (0.80, 2.00)	1.00 (0.78, 1.88)	0.817
	Postoperative	0.40 (0.22, 0.70)	0.52 (0.40, 0.80)	0.076
***P*** value (Pre vs. Post)		***P*** < 0.001	***P*** < 0.001	

BCVA: Best-corrected visual acuity; LogMAR: Logarithm of the minimum angle of resolution

## Discussion

The current study found that anterior capsule polishing can prevent anterior capsule fibrosis after phacovitrectomy without primary IOL implantation. The efficiency is independent of other factors such as type of tamponade and diabetes. Furthermore, anterior capsule polishing promotes reopening of the capsule bag and in-the-bag implantation of IOL in the second stage.

There are several reports on the prevention of anterior capsule fibrosis after phacoemulsification with IOL implantation [11, 12], but not for phacovitrectomy without IOL implantation, which is more challenging as no barrier to prevent the adhesion of anterior and posterior capsule. The other reported preventive techniques include larger capsulorhexis size [13], anterior capsule Nd: YAG laser relaxing incisions [14], can-open capsulotomy [15], and lens epithelial cells cleanup [16, 17]. Larger capsulorhexis may not be able to cover the edge of IOL and impair the stability of IOL. Anterior capsule Nd: YAG may cause posterior capsule rupture as it adhered with anterior capsule. Can-open capsulotomy may lead to capsule tear during phacoemulsification. Anterior capsule polishing has been shown to prevent anterior capsule fibrosis after phacoemulsification with IOL implantation [16, 17]. In this study, we further verify its efficiency in phacovitrectomy without IOL implantation. The severity of anterior capsule fibrosis is also affected by the material and design of IOL [18, 19]. In this study, since the IOL was not implanted in the first stage operation, therefore the interference of the IOL for fibrous proliferation was eliminated.

Anterior capsule fibrosis is a wound-healing response of the residual lens epithelial cells located underneath the anterior capsule after cataract surgery [11]. Using an irrigation/aspiration tip to polish the anterior lens capsule is an effective technique to remove the residual lens epithelial cells [20] and therefore easily explains the results of the current study. In this study, we introduced a novel 0–36 scoring system to quantify anterior capsular changes. Unlike traditional qualitative grading, our clock-hour-based approach allows for a more precise assessment of the ‘fibrotic burden’ on the capsular bag. However, minimizing fibrosis must be balanced against the risk of ‘Dead Bag Syndrome’ (DBS) caused by excessive LEC depletion. DBS is characterized by a clear but diaphanous and floppy capsule that fails to support the IOL years after surgery. Since LECs are essential for capsular structural integrity, extensive polishing may inadvertently trigger LECs depletion and capsule weakening [21]. Therefore, a balanced approach is necessary to mitigate fibrosis without compromising long-term mechanical stability.

This study also found that diabetes and silicone oil tamponade are two independent risk factors for anterior capsule fibrosis. Some studies found that diabetes increases the risk of anterior capsule contraction [22]. The explanation may be due to higher inflammatory reactions in patients with diabetes. However, dedicated studies specifically investigating the correlation between silicone oil tamponade and anterior capsule fibrosis are limited. Patients with silicone oil tamponade may have more severe posterior eye segment conditions and a longer time of prone posture, which may promote inflammation at the anterior segment and the proliferation of lens epithelial cells.

This study provides a simple technique to prevent anterior capsule fibrosis after phacovitrectomy without IOL implantation. This can help to improve the postoperative visualization of the retina and management of retinal diseases, which usually need long time follow-up and multiple management after phacovitrectomy. Another clinical significance of this study is the facilitation of the reopening of the capsular bag and implantation of IOL in the bag, which would help to improve the postoperative visual quality and prevent postoperative complications. In another study, we showed that the mean IOL tilt and decentration of secondary in-the-bag intraocular lens (IOL) implantation following phacovitrectomy with anterior capsule polishing was comparable with the group of primary IOL implantation [23].

We also recognize some limitations of the current study. First, this is a retrospective study and there may be bias in patients’ selection. There is an imbalance in sample sizes between the polishing and non-polishing groups. Further studies with randomized control design are needed to provide stronger evidence for the current finding. Second, the severity of anterior capsule fibrosis was graded subjectively. We used two independent assessors, their assessment was in a blind manner, and the intraclass correlation was high. Third, the current study only found that IOL can be implanted in the bag in most cases receiving anterior capsule polishing. The tilt and decentration of IOL in these cases need further investigation.

In conclusion, 360-degree anterior capsule polishing is associated with less anterior capsule fibrosis after phacovitrectomy without IOL implantation. It is also associated with higher chances of the re-opening of capsular bag and in-the-bag implantation of IOL at the second stage.

## Supplementary Information

Below is the link to the electronic supplementary material.

Supplementary Material 1

## Data Availability

The datasets generated and/or analysed during the current study are not publicly available due to the fact that they contain private information of research participants but are available from the corresponding author on reasonable request.

## References

[1] Villegas VM, Gold AS, Latiff A, Wildner AC, Ehlies FJ, Murray TG. Phacovitrectomy Dev Ophthalmol. 2014;54:102–7. 25196758 10.1159/000360455

[2] Seider MI, Michael Lahey J, Fellenbaum PS. Cost of phacovitrectomy versus vitrectomy and sequential phacoemulsification. Retina. 2014;34(6):1112–5. 24608671 10.1097/IAE.0000000000000061

[3] Moussa G, Sachdev A, Mohite AA, Hero M, Ch’ng SW, Andreatta W. Assessing refractive outcomes and accuracy of biometry in phacovitrectomy and sequential operations in patients with retinal detachment compared with routine cataract surgery. Retina. 2021;41(8):1605–11. 33394963 10.1097/IAE.0000000000003092

[4] Wu K, Zong Y, Yu J, Fang W, Jiang C, Xu G. Secondary in-the-bag intraocular lens implantation in aphakic eyes after vitrectomy and silicone oil tamponade for rhegmatogenous retinal detachment. Retina. 2023;43(8):1408–12. 33003173 10.1097/IAE.0000000000002987

[5] Luo L, Lin H, Chen W, Wang C, Zhang X, Tang X, et al. In-the-bag intraocular lens placement via secondary capsulorhexis with radiofrequency diathermy in pediatric aphakic eyes. PLoS ONE. 2013;8(4):e62381. 23638058 10.1371/journal.pone.0062381 PMC3634760

[6] Grewal DS, Basti S. Modified technique for removal of Soemmerring ring and in-the-bag secondary intraocular lens placement in aphakic eyes. J Cataract Refract Surg. 2012;38(5):739–42. 22520301 10.1016/j.jcrs.2012.02.023

[7] Liu YJ, Zhang WW, Chen FF, He ZF, Xie ZG. Reopening the severely contracted lens capsular bag post-phacovitrectomy by injecting OVD and removing the fibrous membranous material. J Cataract Refract Surg. 2021;47(12):e66–9. 33929794 10.1097/j.jcrs.0000000000000675

[8] Biswas P, Batra S, Commentary. Anterior capsule polishing: The present perspective. Indian J Ophthalmol. 2020;68(5):785–6. 32317446 10.4103/ijo.IJO_2088_19 PMC7350444

[9] Bolz M, Menapace R, Findl O, Sacu S, Buehl W, Wirtitsch M, et al. Effect of anterior capsule polishing on the posterior capsule opacification-inhibiting properties of a sharp-edged, 3-piece, silicone intraocular lens: three- and 5-year results of a randomized trial. J Cataract Refract Surg. 2006;32(9):1513–20. 16931265 10.1016/j.jcrs.2006.04.020

[10] Han MY, Yu AH, Yuan J, Cai XJ, Ren JB. Effect of anterior capsule polish on visual function: A meta-analysis. PLoS ONE. 2019;14(1):e0210205. 30620750 10.1371/journal.pone.0210205 PMC6324835

[11] Shah SK, Praveen MR, Kaul A, Vasavada AR, Shah GD, Nihalani BR. Impact of anterior capsule polishing on anterior capsule opacification after cataract surgery: a randomized clinical trial. Eye (Lond). 2009;23(8):1702–6. 19079142 10.1038/eye.2008.355

[12] Baile R, Sahasrabuddhe M, Nadkarni S, Karira V, Kelkar J. Effect of anterior capsular polishing on the rate of posterior capsule opacification: A retrospective analytical study. Saudi J Ophthalmol. 2012;26(1):101–4. 23960976 10.1016/j.sjopt.2010.11.006 PMC3729577

[13] Rossi T, Ceccacci A, Testa G, Ruggiero A, Bonora N, D’Agostino I, et al. Influence of anterior capsulorhexis shape, centration, size, and location on intraocular lens position: finite element model. J Cataract Refract Surg. 2022;48(2):222–9. 34117178 10.1097/j.jcrs.0000000000000711 PMC8845527

[14] Hayashi K, Yoshida M, Hirata A, Hayashi H. Anterior capsule relaxing incisions with neodymium:YAG laser for patients at high-risk for anterior capsule contraction. J Cataract Refract Surg. 2011;37(1):97–103. 21067893 10.1016/j.jcrs.2010.07.027

[15] Davison JA. Capsule contraction syndrome. J Cataract Refract Surg. 1993;19(5):582–9. 8229711 10.1016/s0886-3350(13)80004-1

[16] Gao Y, Dang GF, Wang X, Duan L, Wu XY. Influences of anterior capsule polishing on effective lens position after cataract surgery: a randomized controlled trial. Int J Clin Exp Med. 2015;8(8):13769–75. 26550324 PMC4613009

[17] Liu B, Zhang L, Fang S. Efficacy and safety of anterior capsule polishing in cataract patients: a meta-analysis. Am J Transl Res. 2023;15(5):3662–73. 37303670 PMC10250971

[18] Corydon C, Lindholt M, Knudsen EB, Graakjaer J, Corydon TJ, Dam-Johansen M. Capsulorhexis contraction after cataract surgery: comparison of sharp anterior edge and modified anterior edge acrylic intraocular lenses. J Cataract Refract Surg. 2007;33(5):796–9. 17466850 10.1016/j.jcrs.2007.01.020

[19] Sacu S, Menapace R, Findl O. Effect of optic material and haptic design on anterior capsule opacification and capsulorrhexis contraction. Am J Ophthalmol. 2006;141(3):488–93. 16490494 10.1016/j.ajo.2005.10.041

[20] Mathey CF, Kohnen TB, Ensikat HJ, Koch HR. Polishing methods for the lens capsule: histology and scanning electron microscopy. J Cataract Refract Surg. 1994;20(1):64–9. 8133483 10.1016/s0886-3350(13)80046-6

[21] Culp C, Qu P, Jones J, Fram N, Ogawa G, Masket S, et al. Clinical and histopathological findings in the dead bag syndrome. J Cataract Refract Surg. 2022;48(2):177–84. 34261985 10.1097/j.jcrs.0000000000000742

[22] Hayashi Y, Kato S, Fukushima H, Numaga J, Kaiya T, Tamaki Y, et al. Relationship between anterior capsule contraction and posterior capsule opacification after cataract surgery in patients with diabetes mellitus. J Cataract Refract Surg. 2004;30(7):1517–20. 15210231 10.1016/j.jcrs.2003.11.045

[23] Zheng C, Huang B, Liang H, Liao X, Grzybowski A, Chen H. Outcomes of secondary in-the-bag intraocular lens implantation following phacovitrectomy. Int Ophthalmol. 2025;46(1):40. 41405768 10.1007/s10792-025-03913-8

